# High-Resolution Imaging of a Single Gliding Protofilament of Tubulins by HS-AFM

**DOI:** 10.1038/s41598-017-06249-1

**Published:** 2017-07-21

**Authors:** Jakia Jannat Keya, Daisuke Inoue, Yuki Suzuki, Toshiya Kozai, Daiki Ishikuro, Noriyuki Kodera, Takayuki Uchihashi, Arif Md. Rashedul Kabir, Masayuki Endo, Kazuki Sada, Akira Kakugo

**Affiliations:** 1 Graduate School of Chemical Sciences and Engineering, Hokkaido University, Sapporo, 060-0810 Japan; 2 Faculty of Science, Hokkaido University, Sapporo, 060-0810 Japan; 3 Frontier Research Institute for Interdisciplinary Sciences, Tohoku University, Aramakiaza Aoba 6-3, Aoba-ku, Sendai, 980-8578 Japan; 4 Department of Physics, Kanazawa University, Kakuma-machi, Kanazawa, 920-1192 Japan; 5 Bio-AFM Frontier Research Center, Kanazawa University, Kanazawa, 920-1192 Japan; 6 Institute for Integrated Cell-Material Sciences, Kyoto University, Kyoto, 606-8501 Japan

## Abstract

*In vitro* gliding assay of microtubules (MTs) on kinesins has provided us with valuable biophysical and chemo-mechanical insights of this biomolecular motor system. Visualization of MTs in an *in vitro* gliding assay has been mainly dependent on optical microscopes, limited resolution of which often render them insufficient sources of desired information. In this work, using high speed atomic force microscopy (HS-AFM), which allows imaging with higher resolution, we monitored MTs and protofilaments (PFs) of tubulins while gliding on kinesins. Moreover, under the HS-AFM, we also observed splitting of gliding MTs into single PFs at their leading ends. The split single PFs interacted with kinesins and exhibited translational motion, but with a slower velocity than the MTs. Our investigation at the molecular level, using the HS-AFM, would provide new insights to the mechanics of MTs in dynamic systems and their interaction with motor proteins.

## Introduction

Microtubule (MT)-kinesin, a cytoskeletal component, plays crucial roles in cell through its various spatial and mechanical functions^1–6^. This biomolecular motor system actively participate in cell contractility^4^, development and maintenance of cell polarity^7^ and also in a number of intracellular events such as intracellular transport, regulation of cell morphology and cell mechanics^8, 9^. *In vitro* gliding assay, where kinesins are adhered to a substrate and MTs are propelled by the kinesins through consumption of adenosine triphosphate (ATP)^5^, has been a useful means in unveiling physiological and chemo-mechanical characteristics of this biomolecular motor system^10–12^. Based on the *in vitro* gliding assay nowadays the MT-kinesin system is finding applications for nanotransport, detection, sensing, imaging, etc.^13, 14^. In those works, observation of MTs in the *in vitro* gliding assay has been mainly dependent on the employment of optical microscopes, limited resolution of which often hinders detail exploration and fails to provide adequate information from the gliding assay. In this work, we performed *in vitro* gliding assay of MTs on lipid bilayer and monitored the gliding MTs and PFs of tubulins under a high speed atomic force microscope (HS-AFM). Moreover, utilizing the advantage of the HS-AFM in monitoring a specimen at the molecular level^15–17^, we for the first time directly observed splitting of gliding MTs into single PFs. Upon splitting, the motile MTs were found to abruptly change their direction of movement. The split single PFs also interacted with kinesins and exhibited translational motion like the intact MTs, but with relatively lower velocity. Recently structural disintegration of MTs in the *in vitro* gliding assay has started to draw attention where, based on the investigations under fluorescence microscopy, wear or breakage of MTs into smaller parts^18^ or protofilament bundles (PFBs)^19^ were reported. Using the HS-AFM our direct observation at the molecular level provides new insights to the structural degradation of MTs in an *in vitro* gliding assay. This work would contribute to our current understanding of the mechanics of MTs in dynamic systems, and at the same time would help promote sustainable applications of biomolecular motor systems in synthetic world^20–22^.

## Results and Discussion

### Observation of gliding MTs and tubulin PFs on a kinesin coated substrate

**Figure 1:**
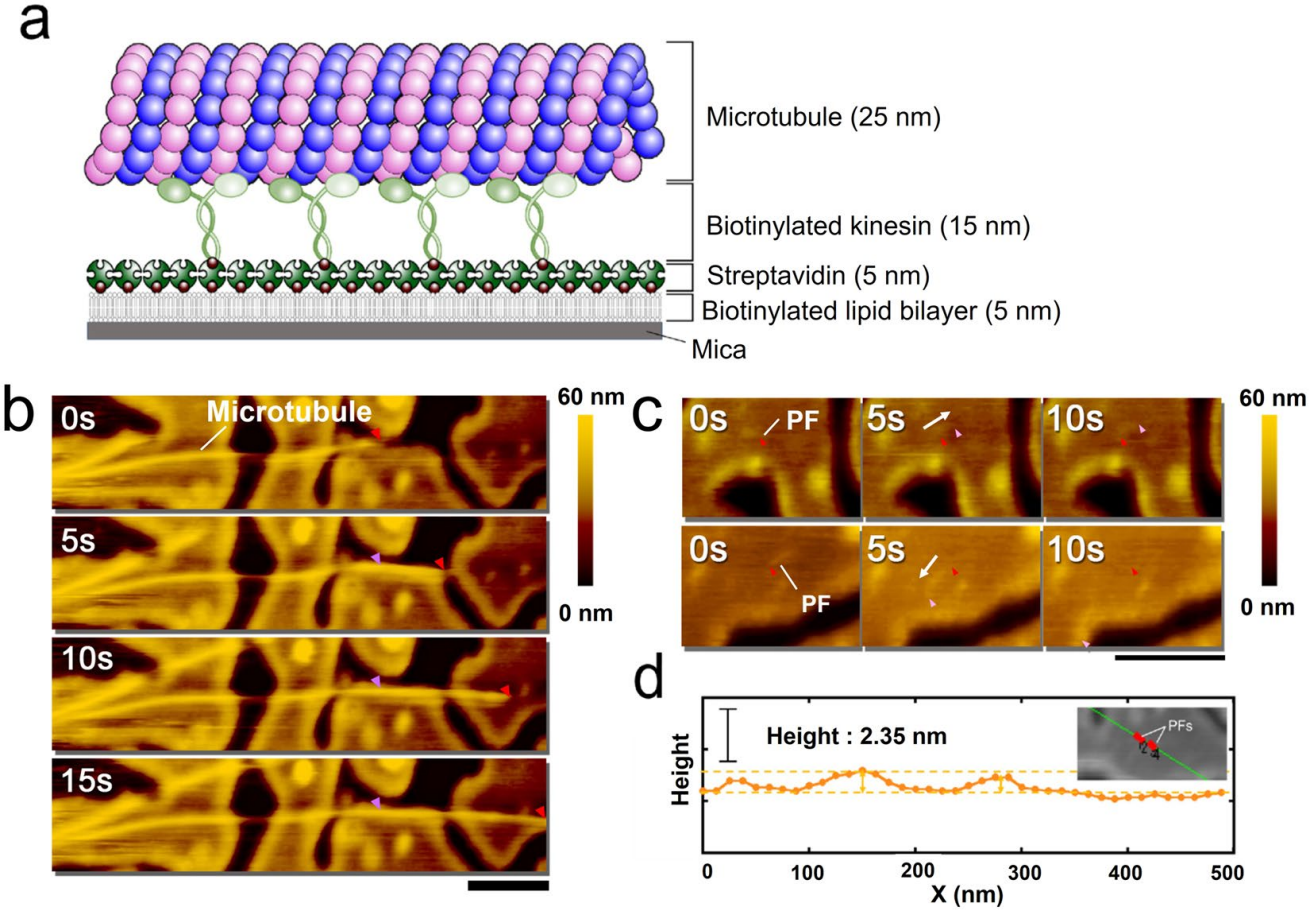
HS-AFM observation of gliding MTs. (**a**) Schematic illustration of *in vitro* gliding assay of MTs on kinesins fixed to a mica supported lipid bilayer through streptavidin/biotin interaction. (**b**) Time lapse images showing gliding motion of a MT on the kinesin coated lipid surface. Scale bar: 500 nm, frame rate: 0.2 s/frame. (**c**) Observation of a gliding PF by using HS-AFM. Scale bar: 500 nm, frame rate: 0.2 s/frame. In (**b**) and (**c**), the pink/red arrows indicate change of position of gliding MT or short PF with time and white arrows show their moving direction. (**d**) Height profile of gliding PFs obtained from HS-AFM image as shown in the inset. Red points in the insets show two PFs.

**Figure 2:**
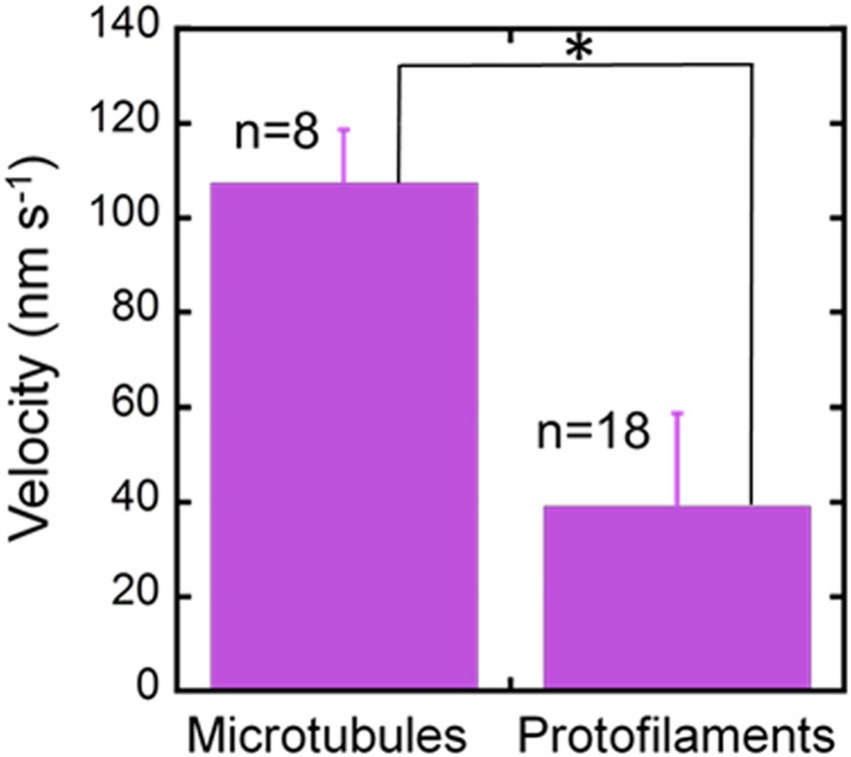
Comparison between velocity of MTs and PFs of tubulins. While gliding on a kinesin coated substrate tubulin PFs moved with a slower velocity than the MTs. Error bar: standard deviation. Velocity differences between the two values are statistically significant. (p < 10^–4^).

We monitored gliding MTs and PFs of tubulins under HS-AFM by demonstrating an *in vitro* gliding assay of MTs on kinesins fixed to a streptavidin coated lipid bilayer on mica (Fig. 1a). The mica-supported biotinylated lipid bilayer was prepared as described in a previous report^23^. In brief, first unilamellar vesicles were prepared from 1,2-Dihexadecanoyl-*sn*-glycero-3-phosphocholine (DPPC), 1,2-Dipalmitoyl-3-trimethylammonium-propane (DPTAP) and 1,2-dipalmitoyl-sn-glycero-3- phosphoethanolamine-N-(cap biotinyl) (biotin-cap DPPE) (see the experimental section for detail). The vesicles were deposited to freshly cleaved mica disks followed by the addition of streptavidin to the lipid bilayer surface. It was observed that upon application of the streptavidin solution, the streptavidin molecules started to form small crystal islands on the biotinylated lipid bilayer (Fig. S1). After decorating the lipid bilayer with streptavidin, biotinylated kinesin solution was added onto the surface. Subsequently, taxol-stabilized MTs were applied and next by adding ATP, the motility of MTs on the kinesin coated substrate was initiated. We monitored the motility of the MTs by HS-AFM, which allowed real time imaging of the motile MTs with a high resolution. As mentioned above, the surface was divided into small islands after addition of streptavidin creating an inhomogeneous surface. As the distance between two islands was much smaller than the length of MTs, we continued the observation of motility of MTs on such surface. The motility of MTs over kinesin coated lipid bilayer substrate can be observed from the images showing their displacement with time (Fig. 1b). While monitoring the gliding MTs under HS-AFM, we also observed smaller and thinner filaments, as shown in Fig. 1c (n~30), with length of 80 ± 30 nm (mean ± S.D.) (Fig. S2) and height (~2.3 nm) (Fig. 1d), gliding around the MTs (Movie S1). From the height profile the smaller and thinner filaments appear to be protofilaments (PFs) of tubulins. The PFs might have been produced during MT polymerization but did not participate in the formation of MTs^24^. While the MTs were moving with a velocity of ~100 nm s^−1^ (n = 8), the PFs were moving with slower velocity (~40 nm s^−1^) (n = 18) (Fig. 2). This difference in velocity is in agreement to a previous report where a difference in velocity between MTs and protofilament bundles (PFBs) was observed under fluorescence microscope^19^. Such difference in velocity of MTs and PFs might be related to the fluctuation of kinesins on the surface, and consequent difference in the extent of force production by the kinesins at MTs or PFs^25^.

An important observation in the present experimental system is the relatively slower velocity of MTs on the lipid layer, compared to that observed in a conventional gliding assay on glass. To investigate the origin of the low velocity of MTs, we performed gliding assay of MTs by changing the substrate and monitored the MTs under HS-AFM. We demonstrated an *in vitro* gliding assay on collodion (nitrocellulose) coated mica substrate which is a hydrophobic surface^26^ (see experimental section). The velocity of MTs was found ~590 nm s^−1^ on the collodion coated mica substrate, which is very close to the velocity of MTs on glass observed in a conventional gliding assay under fluorescence microscope^27^. This result suggests that the slow movement of MTs on lipid bilayer coated mica substrate might be related to the nature of substrate which can affect the specific binding of motor protein on the surface. This was further confirmed when an *in vitro* gliding assay was performed on lipid coated mica substrate prepared from a lipid and composition other than used above. Here we prepared lipid layer on mica using vesicles prepared from 1,2-dioleoyl-*sn*-glycero-3-phosphocholine (DOPC), 1,2-dioleoyl-*sn-*glycero-3-phospho-L-serine (DOPS) and 1,2-dioleoyl-*sn*-glycero-3-phosphoethanolamine-N-(cap biotinyl) (biotin-cap DOPE)^28^ (see the experimental section for detail). On this lipid layer the velocity of MTs was ~150 nm s^−1^ (n = 50), which is slightly higher than that observed for the DPPC/DPTAP/biotin-cap DPPE system. Collectively these results confirm the effect of substrate on the velocity of MTs, suggesting the mechanical process of scanning during observation under HS-AFM not to be the main factor behind the slow velocity of MTs. It should be noted that, while preparing lipid layer using DOPC/DOPS/biotin-cap DOPE lipid system at the prescribed composition (see experimental section), we observed noticeable difference in the topography of streptavidin crystals compared to that for DPPC/DPTAP/biotin-cap DPPE system. While there was an incomplete and fractionate appearance of the streptavidin crystals for the DPPC/DPTAP/biotin-cap DPPE system (Fig. S1), we obtained a much uniform layer of streptavidin crystals (Fig. S3a, S3b) for the DOPC/DOPS/biotin-cap DOPE system. On such a uniform crystal layer of streptavidin, we were able to observe the anchored single kinesins using the HS-AFM (Fig. S4a) with a density ~1200 µm^−2^ (Fig. S4b, Movie S2). The density of kinesins on the lipid layer is found much lower than that on a glass surface^29^. On this lipid layer we observed MTs, confirmed from their height profile (23 ± 1 nm) (Fig. S5), which also exhibited motility (Movie S3). Despite the formation of homogeneous streptavidin crystal layer on the DPPC/DPTAP/biotin-cap DPPE system, the velocity of MTs remained quite slow (~150 nm s^−1^, n = 50), which could be attributed to the low density of kinesin on the streptavidin/lipid surface. Similar to the DPPC/DPTAP/biotin-cap DPPE system difference in velocity between PFs (~60 nm s^−1^, n = 20) and MTs was also observed for the DOPC/DOPS/biotin-cap DOPE system. The path length of PFs was found to be much smaller than MTs and they showed pausing events during their motility (Fig. S6).

**Figure 3:**
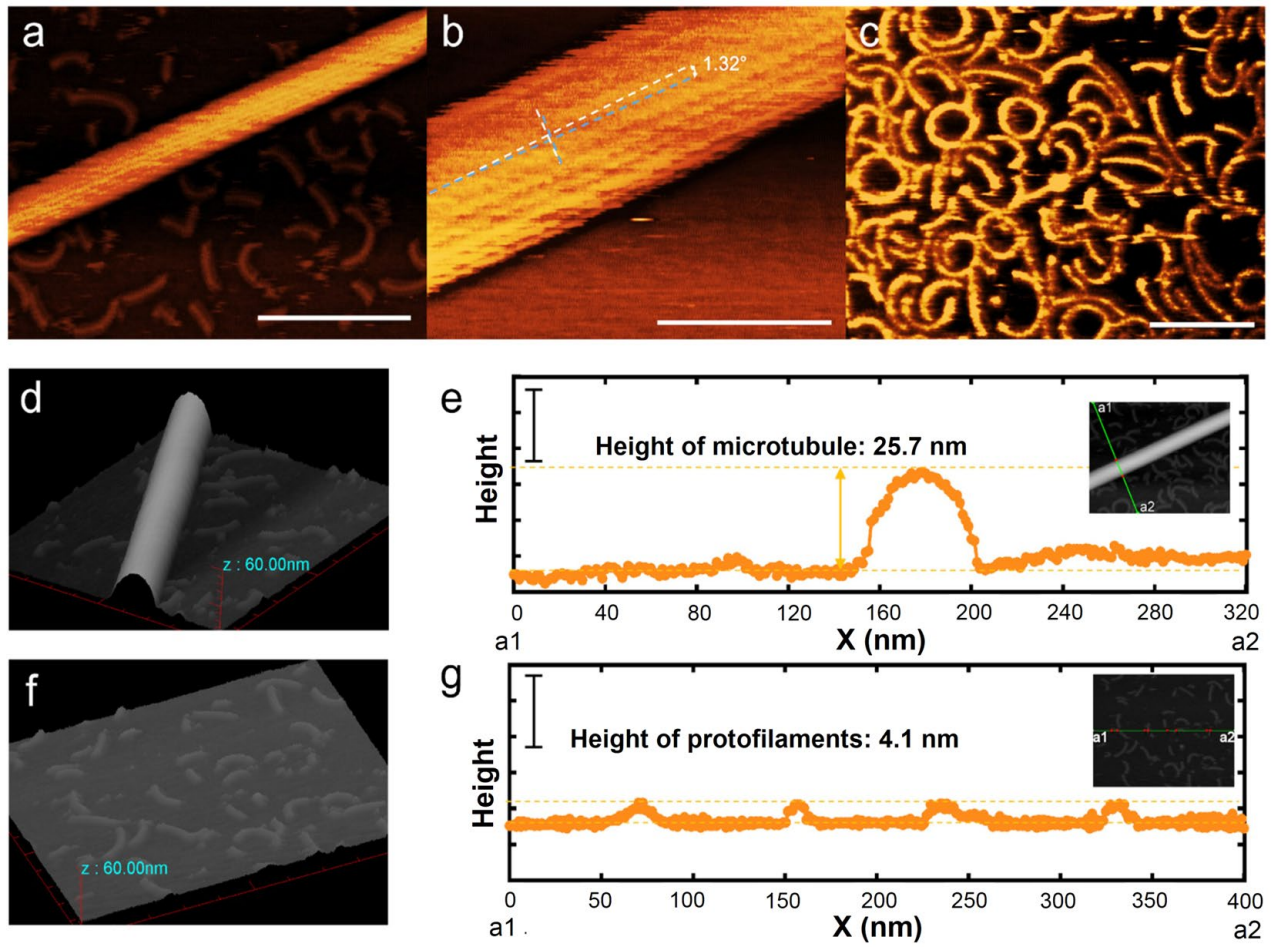
HS-AFM images of MT and PFs of tubulins. (a, b) HS-AFM images of a paclitaxel stabilized MT. Scale bars: (**a**) 250 nm, (**b**) 50 nm and frame rate: 0.2 s/frame. In (**b**) white dotted line indicates the longitudinal axis of the MT and blue dotted line shows direction of the PFs. (**c**) HS-AFM images of PFs of tubulins. Scale bar 100 nm and frame rate: 0.2 s/frame. Topographic image and height profile of a MT (d, e) and PFs (f, g) obtained from HS-AFM. 3D image of a MT and PFs was obtained using HS-AFM.

Generally, taxol-stabilized PFs are known to form curled structures as reported in a previous AFM study^24^. In fact, bundles of PFs form ring shaped structure when detaching from the MTs^19^. However, in our experiment, we didn’t observe such curled shape of single PFs during gliding on the surface. Usually, when a PF starts to be bent, kinesin binding surface of the PF goes inside the PF ring and inner part of the MT is exposed to the outside ring. But here if the kinesin binding side of PF is fixed on a flat kinesin coated surface, PF cannot be bent to form ring anymore and should keep straight structure (Fig. S7). On the other hand, if PFs form bundle, it may produce more force to bend PFs. Therefore, single PFs are found to exhibit translational motion, unlike the PFBs which exhibit circular motion on the kinesin coated surface^19^. To confirm further, we imaged MTs and PFs of tubulins fixed to mica substrate through electrostatic interaction (Fig. 3a–g). A magnified HS-AFM image shows helical lattice of MT filaments with a helix angle ~1 degree where the number of observed PFs was 16 (Fig. 3b). Short and thin filaments with length of 50 ± 20 nm (n~60, Fig. S2) and circular, curled and straight conformation were also observed (Fig. 3c). From the topographic image and height profile, the height of a MT filament was obtained as ~25 nm (Fig. 3d, e). On the other hand, for the thinner filaments the height was ~4 nm (Fig. 3f, g), and these findings agree well with the literature^24^. Based on this discussion, we can confer that the gliding thinner filaments observed under HS-AFM in the *in vitro* gliding assay are PFs of tubulins.

### Observation of splitting of gliding MTs into single PFs

**Figure 4:**
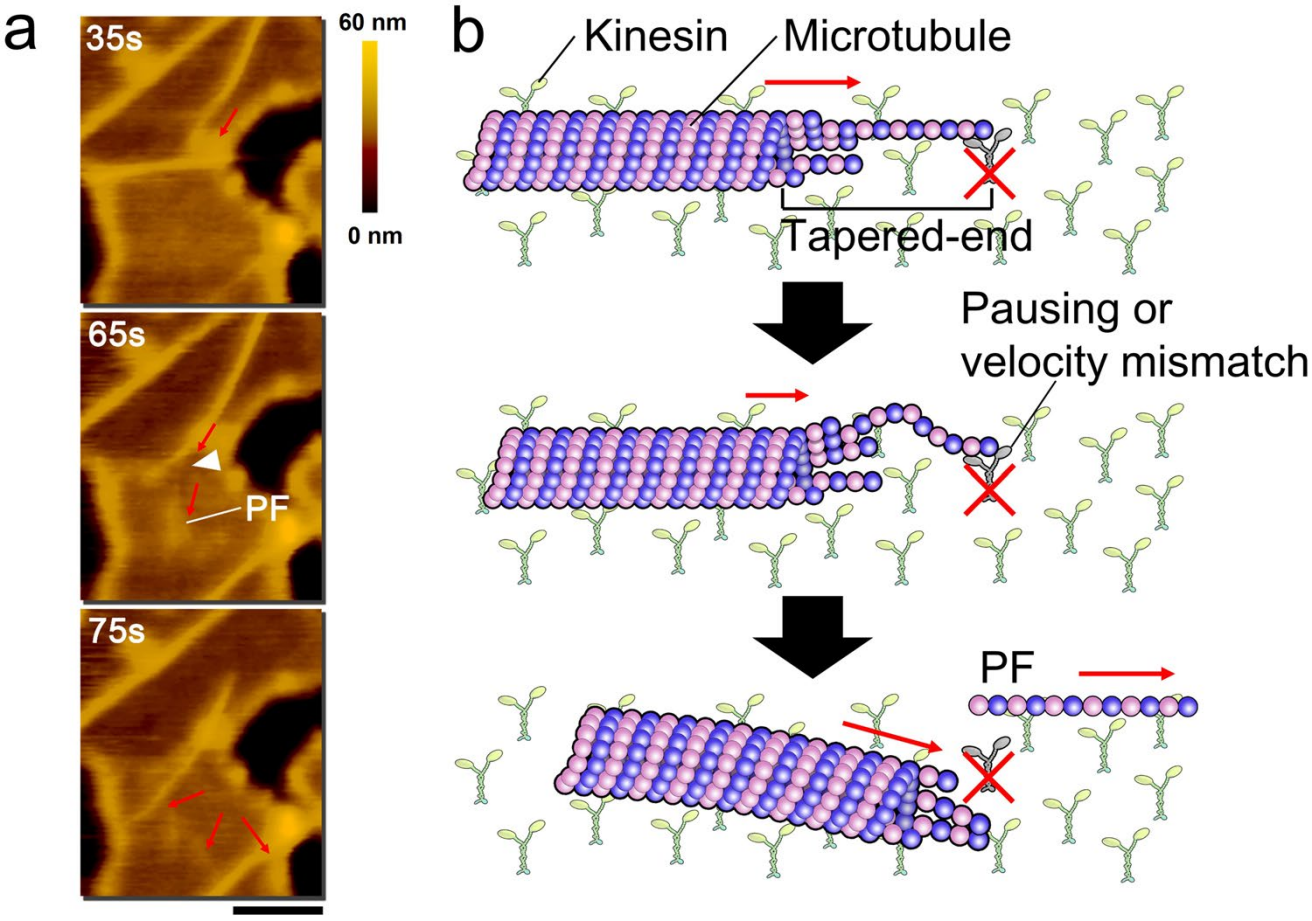
Sudden change in direction of gliding MTs with segregation of tapered PFs from the leading end. (**a**) Time-lapse images showing splitting of a MT at the tapered leading end during gliding motion. The red arrows show moving direction of gliding MTs and PFs separated from leading end of the MT. Scale bar: 500 nm and frame rate: 0.2 s/frame. (**b**) Schematic model showing sudden directional change of MT with segregation of a PF from the tapered leading end of the MT.

Motility of MTs on a kinesin coated substrate has been reported to be associated with molecular wear, breakage or splitting of MTs into PFBs^18, 19^. The broken and split MTs exhibited translational and circular motion respectively on the kinesin coated substrate^18, 19^. Here taking the advantage of high resolution imaging of gliding MTs by HS-AFM, we directly observe the splitting of gliding MTs into single PFs on lipid coated mica substrate (Movie S4, S5). While monitoring the motile MTs by HS-AFM, we observed that the MTs suddenly changed their moving direction after a short pause. Such abrupt change in direction of motion of the MTs was also observed under fluorescence microscope, although the reason has remained obscure. Our high resolution imaging by HS-AFM revealed that after pausing, a gliding MT was split into two fragments (Fig. 4a). This result suggests that, the sudden change in direction of motile MTs is related to such splitting of the MTs. Probably, the leading end of a MT has tapered-part^30^ (Fig. S8) and when PFs at the tapered leading end of MT collides with dead kinesins or some obstacles, the PFs might be bent, and being pushed by the lagging part of the MT. Finally, the tapered PFs are broken and separated from the leading end of MTs. Consequently, MTs might change their moving direction due to the buckling force from the PF (Fig. 4b). Additionally, taking into account the difference in the velocity between PFs and MTs, PFs tapered at the leading end of a MT might also cause bending of tapered-PFs due to the mismatch of the velocity and subsequently causing directional change of MTs. Thus, from our observation under HS-AFM, we revealed that splitting of PFs from the leading end of MTs influences the motility of MTs. However, we can see splitting of MTs into PFs on the lipid bilayer surface with some fractionate appearance of streptavidin crystals (DPPC/DPTAP/biotin-cap DPPE) (Fig. 4). We also observed such splitting on the uniform large areas of streptavidin crystals using the other lipid bilayer (DOPC/DOPS/biotin-cap DOPE) surface (Movie S5) which indicates that incomplete or fractionate appearance of streptavidin crystals might have no considerable effect on the splitting of gliding MTs into PFs. The PF split from the motile MTs showed translational motion, which is in contrary to the circular motion exhibited by PFBs^19^.

## Conclusion

In conclusion, exploiting the advantage of high resolution imaging by HS-AFM at molecular scale, we have successfully observed gliding motion of MTs and single PFs in an *in vitro* gliding assay. Moreover, splitting of the gliding MTs into single PFs has also been observed directly for the first time, which can account for the sudden directional change of the gliding MTs in the gliding assay. Our investigation thus provides an answer to the long standing mystery of sudden directional change of gliding MTs in an *in vitro* gliding assay, which has been observed under epi-fluorescence microscopy. From the detail investigation of motile MTs at the molecular level using HS-AFM, detail of the relationship between structure of MTs and their motility behavior would be understandable. High resolution imaging by HS-AFM might be advantageous in the study of MT dynamics or interaction between motor proteins and MTs with defects in their lattice^31–34^ and impact of structural change of MTs by MAPs^35^, motor proteins^36, 37^ or post translational modification^38^ on their functionality in a dynamic system.

## Materials and Methods

### Purification of tubulin and kinesin

Tubulin was purified from porcine brain using a high-concentration PIPES buffer (1 M PIPES, 20 mM EGTA, and 10 mM MgCl_2_; pH adjusted to 6.8 using KOH). The high-concentration PIPES buffer and Brinkley Buffer 80 (BRB80) (80 mM PIPES, 1 mM EGTA, 1 mM MgCl_2_, pH 6.8) were prepared using PIPES from Sigma, and the pH was adjusted using KOH^39^. Kinesin construct consisting of human kinesin (residues 1-465), with an N-terminal histidine tag, and a C-terminal avi-tag were prepared as described in previously published reports by partially modifying the expression and purification methods^40, 41^.

### Preparation of MTs

For preparation of MTs, 56 µM tubulin was incubated at 37 °C for 30 min using polymerization buffer including guanosine-5′-triphosphate (GTP) (80 mM PIPES, 1 mM EGTA, 1 mM MgCl_2_, 1 mM GTP, pH ~6.8). Tubulin and polymerization buffer were mixed at a 4:1 volume ratio. Finally polymerized MTs were stabilized with taxol buffer (80 mM PIPES, 1 mM EGTA, 1 mM MgCl_2_, 10 µM Paclitaxel, 5% DMSO).

### Observation of MTs and PFs on mica surface

1 µM MT was directly deposited on the mica surface and washed with 40 µL taxol buffer including 3 µM paclitaxel to avoid the crystals of taxol during observation and observed by HS-AFM.

### Preparation of streptavidin coated lipid bilayer surface

Lipid system 1: Biotinylated mica-supported lipid bilayers were prepared as described in a previous report^23^. Briefly, solution of 1,2-Dihexadecanoyl-*sn*-glycero-3-phosphocholine (DPPC) in chloroform, 1,2-Dipalmitoyl-3-trimethylammonium-propane (DPTAP) and 1,2-dipalmitoyl-sn-glycero-3- phosphoethanolamine-N-(cap biotinyl) (biotin-cap DPPE) (Avanti Polar Lipids) were mixed at a weight ratio of 0.85:0.05:0.10. The chloroform was then evaporated under a stream of nitrogen gas, and the lipids were redissolved in MilliQ H_2_O at a concentration of 1 mg mL^−1^. Solubilized lipid was diluted to ~0.05 mg mL^−1^ by MilliQ H_2_O and sonicated to obtain small unilamellar vesicles. A drop of the vesicle solution (3 μL) was deposited on freshly cleaved mica disks. After ~3 h of incubation in a sealed container, in which a Kimwipe wetted with MilliQ H_2_O was placed, the surface was rinsed with crystallization buffer 1 (20 mM Tris-HCl, 1 mM EDTA, 10 mM MgCl_2_, pH~7.6). Then, a drop (3 μL) of streptavidin in crystallization buffer 1 (1 μg mL^−1^) was deposited on the bilayer surface. After 30 min of incubation, the surface was rinsed again with crystallization buffer 1 to remove the excess streptavidin.

Lipid system 2: For the DOPC/DOPS/biotin-cap DOPE system, solution of 1,2-dioleoyl-*sn*-glycero-3-phosphocholine (DOPC) in chloroform, 1,2-dioleoyl-*sn-*glycero-3-phospho-L-serine (DOPS) and 1,2-dioleoyl-*sn*-glycero-3-phosphoethanolamine-N-(cap biotinyl) (biotin-cap DOPE) (Avanti Polar Lipids) were mixed at a weight ratio of 7:2:1. The chloroform was then evaporated under a stream of nitrogen gas, and the lipids were redissolved in MilliQ H_2_O at a concentration of 1 mg mL^−1^. Solubilized lipid was diluted to ~0.05 mg mL^−1^ by MilliQ H_2_O and sonicated to obtain small unilamellar vesicles. A drop of the vesicle solution (3 μL) was deposited on freshly cleaved mica disks. After ~3 h of incubation in a sealed container, in which a Kimwipe wetted with MilliQ H_2_O was placed, the surface was rinsed with crystallization buffer 2 (10 mM HEPES-NaOH, 150 mM NaCl, 2 mM CaCl_2_, pH 7.4). Then, a drop (3 μL) of streptavidin in crystallization buffer 2 (1 mg mL^−1^) was deposited on the bilayer surface. After 3 h of incubation, the surface was rinsed again with crystallization buffer 2 to remove the excess streptavidin. For the chemical fixation of the streptavidin crystals, a 10 mM glutaraldehyde-containing crystallization solution 2 was applied and incubated for 5 min. The reaction was quenched using 20 mM Tris buffer mixed in the crystallization buffer 2.

### Preparation of motility assay of MTs on lipid bilayer coated mica substrate

A drop (2 μL) of biotinylated kinesin (6 μM and 200 nM for Lipid system 1 and 2 respectively) in BRB80 with 1 mM DTT (80 mM PIPES, 1 mM EGTA, 1 mM MgCl_2_, 1 mM DTT, pH 6.8) was deposited on the 2D streptavidin crystal surface. After 2~3 min incubation the surface was rinsed with 20 μL BRB80 to remove excess kinesin and then a drop of 1 μM MT solution in BRB80 with 3 μM paclitaxel (80 mM PIPES, 1 mM EGTA, 1 mM MgCl_2_, 1 mM DTT, 3 μM paclitaxel/DMSO; pH 6.8) was deposited on the surface. After 3 min of incubation, the surface was rinsed with 20 μL taxol buffer and imaged by AFM in ~120 μL of the taxol buffer containing 2 mM ATP.

### Preparation of motility assay on the nitrocellulose coated glass surface

2% nitrocellulose (NC) in isoamyl acetate (0.5 µL) was deposited on a freshly cleaved mica surface for 15 min to completely dry. Then 200 nM kinesin-1 (2 µL) in BRB80 with 1 mM DTT (80 mM PIPES, 1 mM EGTA, 1 mM MgCl_2_, 1 mM DTT, pH 6.8) was deposited on the surface for 5 min. After that washed by excess amount of BRB80 with DTT (~100 µL) buffer. 1 mg mL^−1^ casein in BRB80 (2 µL) was deposited on the surface for 3 min. Then rinsed with taxol buffer (BRB80 with 3 µM paclitaxel) (20 µL). 1 µM of MT in taxol buffer was deposited and waited for 5 min. Finally rinsed with taxol buffer ~60 µL and imaged by AFM in ~120 µL of the taxol buffer containing 2 mM ATP.

### HS-AFM imaging

AFM imaging was performed using a tip scan high-speed AFM imaging system (BIXAM, olympus, Tokyo, Japan) that was improved based on a previously developed machine^42^ and laboratory-made high-speed AFM imaging system^15^. Silicon nitride cantilevers (resonant frequency = 0.4–1.0 MHz in water, spring constant = 0.1 Nm^−1^, electron-beam-deposited tip radius <15 nm; Olympus BLAC10EGS-A2). AFM images were analyzed using the AFM Scanning System Software (Olympus, Tokyo, Japan), laboratory made software, Adobe Photoshop CC and ImageJ^43^.

## Electronic supplementary material

Supplementary Information

Movie S1

Movie S2

Movie S3

Movie S4

Movie S5
