# Supplementary material for: High-Resolution Imaging of a Single Gliding Protofilament of Tubulins by HS-AFM
Source: Sci Rep. 2017 Jul 21;7:6166. doi: 10.1038/s41598-017-06249-1 (PMC5522458; doi:10.1038/s41598-017-06249-1)
Supplement: Supplementary file 1 — Supplementary Information [file 41598_2017_6249_MOESM1_ESM.pdf]

High-Resolution Imaging of a Single Gliding Protofilament of Tubulins by HS-AFM

Jakia Jannat Keya [†], *Daisuke Inoue* [†], *Yuki Suzuki, Toshiya Kozai, Daiki Ishikuro,*

Noriyuki Kodera, Takayuki Uchihashi, Arif Md. Rashedul Kabir, Masayuki Endo,

Kazuki Sada and Akira Kakugo [*]

Supplementary Information

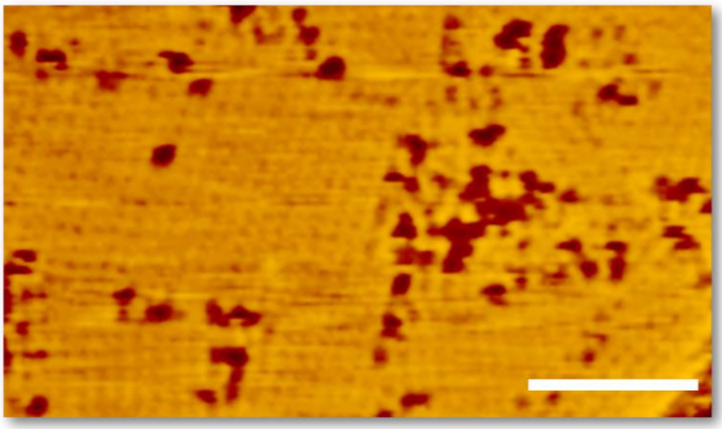

Figure S1 | HS-AFM images of two dimensional crystals of streptavidin. HS-AFM image of surface of the lipid membrane coated with streptavidin crystals. Scale bar: 50 nm, frame rate: 0.2 s/frame.

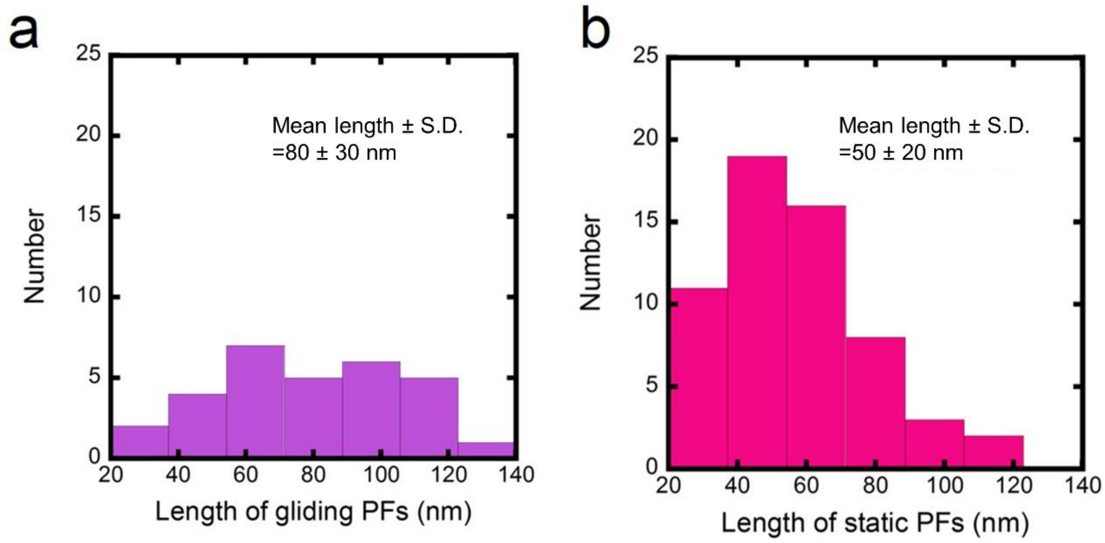

Figure S2 | Comparison of length distribution of tubulin PFs observed in (a) motile and (b) static condition. Images of PFs were captured when the PFs were gliding on kinesins fixed to lipid bilayer coated mica substrate (a) and fixed to a mica substrate through electrostatic interaction (b).

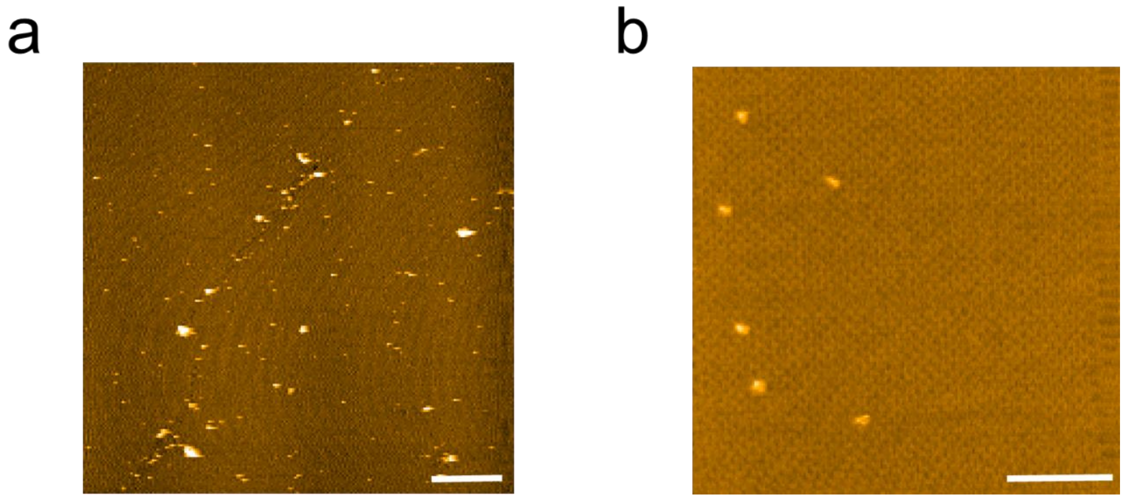

Figure S3 | HS-AFM images of two dimensional crystals of streptavidin. (a) and (b). HS-AFM image of larger area of streptavidin 2D crystal on lipid membrane surface. Scale bar: 200 nm, frame rate: 1.0 s/frame (a) and magnified image of center part of image a. Scale bar: 40 nm (b).

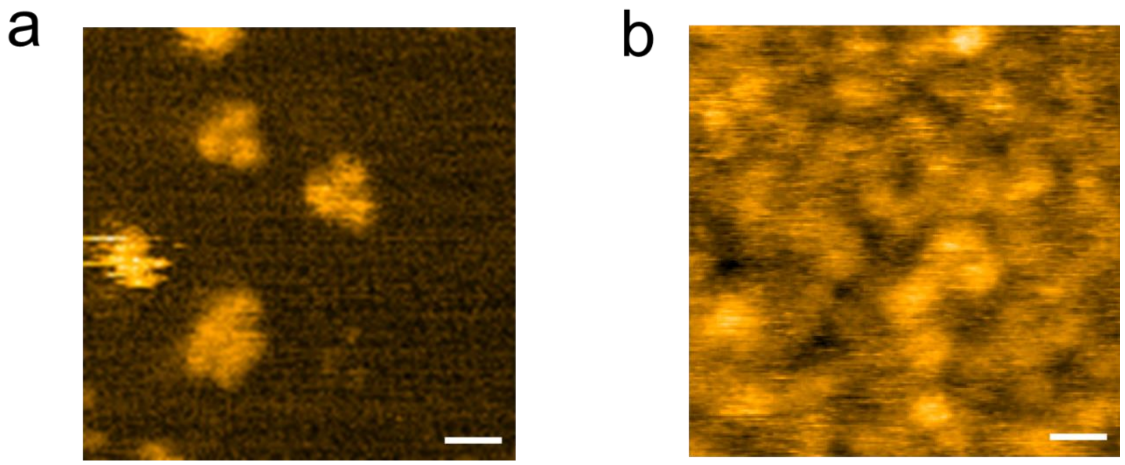

Figure S4 | Motor protein kinesin on streptavidin 2D crystal surface (a) and (b). Bright spots are kinesin with head groups. Scale bar: 20 nm, frame rate: 1.0 s/frame (a) and (b).

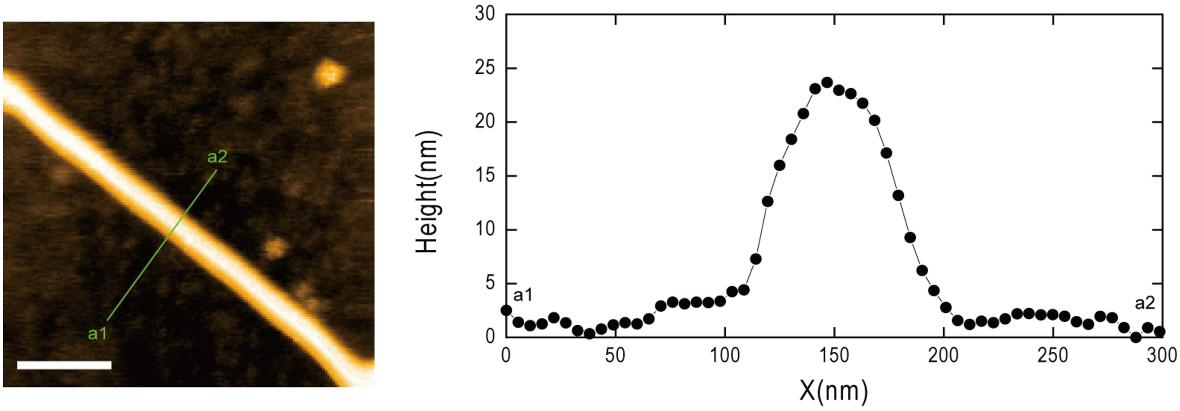

Figure S5 | Topographic image of MT on streptavidin 2D crystal coated lipid bilayer surface fixed by kinesin (left) and its height profile (right). Scale bar: 200 nm, frame rate: 1.0 s/frame.

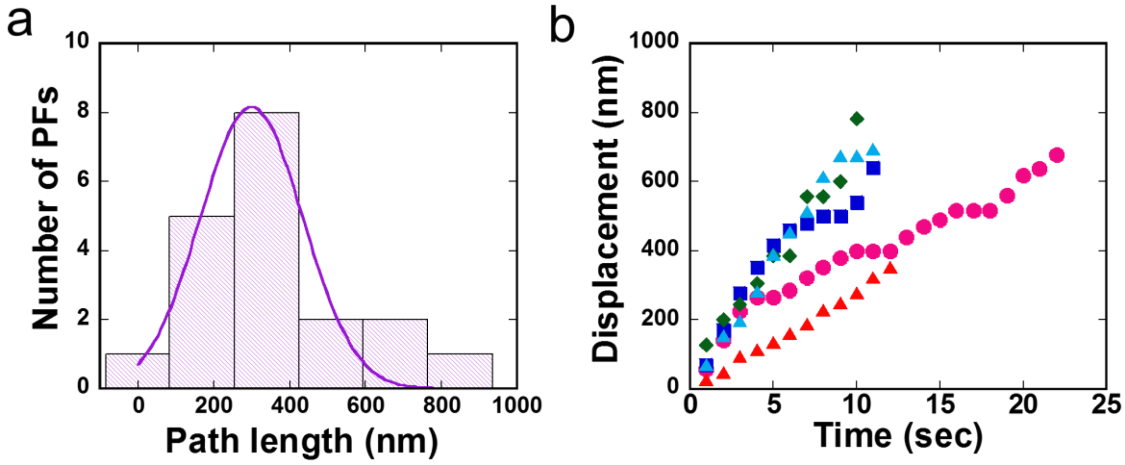

Figure S6 | Path length of gliding PFs (a) and (b). Distribution of path length of gliding PFs. Mean=300± 190, R^2=0.87. n=20 (a) and typical displacement of PFs with time. n=5 (b).

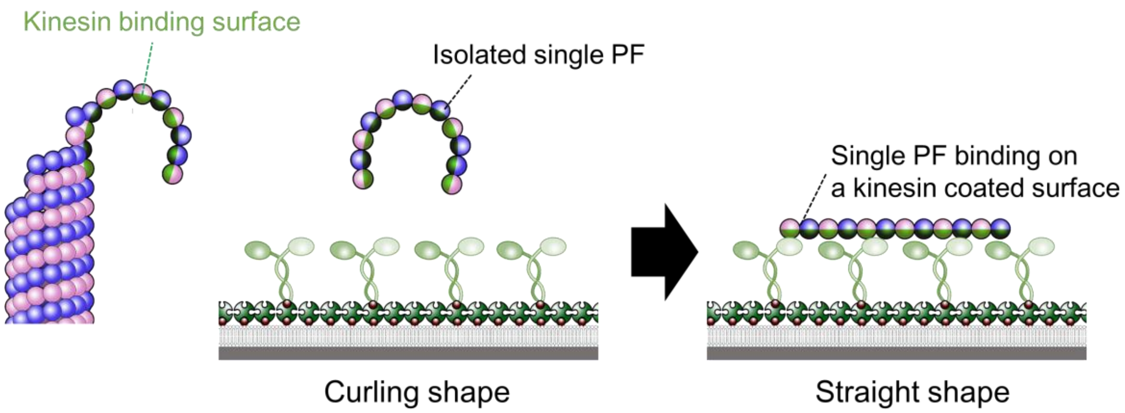

Figure S7 | Schematic diagram showing binding of a PF of tubulins to kinesin coated surface. The PFs of tubulin showed translational motion on kinesins.

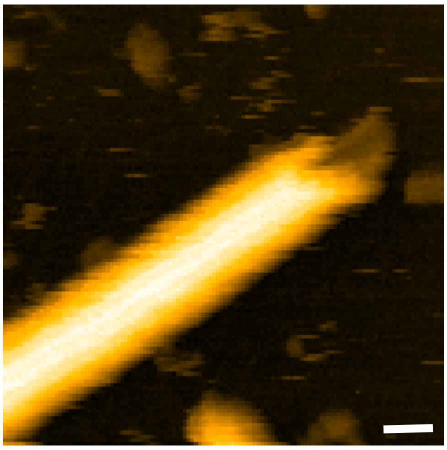

Figure S8 | HS-AFM image of a MT with a tapered end. The bright cylindrical object is MT. At the very end of the MT tapered end is observed showing PFs. Scale bar: 20 nm, frame rate: 1.0 s/frame.

Movie captions

Supplementary Movie S1. Motility of tubulin PFs and MT on kinesins fixed to mica supported streptavidin coated lipid bilayer (DPPC/DPTAP/biotin-cap DPPE) surface. Scan area: 4000 × 3000 nm^2. Observation period: 530 s.

Supplementary Movie S2. Saturated surface of kinesin on mica supported streptavidin coated lipid bilayer (DOPC/DOPS/biotin-cap DOPE) surface**.** Scan area: 150 × 150 nm^2. Observation period: 25 s.

Supplementary Movie S3. Motility of MTs on mica supported streptavidin coated lipid bilayer (DOPC/DOPS/biotin-cap DOPE) surface**.** Scan area: 2500 × 2500 nm^2. Observation period: 180 s.

Supplementary Movie S4. Splitting of PFs from gliding MT and change of direction of MT on kinesins fixed to mica supported streptavidin coated lipid bilayer (DPPC/DPTAP/biotin-cap DPPE) surface. Scan area: 1500 × 2000 nm^2. Observation period: 80 s.

Supplementary Movie S5. Splitting of PF from gliding MT and its motility on mica supported streptavidin coated lipid bilayer (DOPC/DOPS/biotin-cap DOPE) surface**.** Scan area: 1000 × 500 nm^2. Observation period: 75 s.
